# The Impact of ABO Incompatibility on the Outcomes of Hematopoietic Stem Cell Transplantation: A Single-Center Study From Pakistan

**DOI:** 10.7759/cureus.45442

**Published:** 2023-09-18

**Authors:** Munira Borhany, Muhammad Shujat Ali, Zainab Ghias, Madiha Abid, Sidra Zafar, Tahir Shamsi

**Affiliations:** 1 Department of Clinical Hematology and Bone Marrow Transplantation, National Institute of blood Diseases, Karachi, PAK; 2 Department of Clinical Hematology and Bone Marrow Transplantation, National Institute of Blood Diseases, Karachi, PAK; 3 Research, National Institute of Blood Diseases, Karachi, PAK

**Keywords:** blood transfusion, allogenic hematopoietic stem cell transplantation, mismatch, compatibility, abo incompatibility

## Abstract

Background and objective

Allogeneic hematopoietic stem cell transplantation (alloHSCT) provides curative treatment for several hematological illnesses. In this study, we evaluated the impact of ABO compatibility and incompatibility on outcomes and complications related to hematopoietic stem cell transplantation (HSCT) performed for various hematological disorders at our center.

Methodology

This was a retrospective, single-center, cohort study in which patients were categorized according to the ABO match and mismatch status. The mismatch group was further subcategorized into major, minor, and bidirectional groups.

Results

A total of 117 patients underwent alloHSCT, out of which 82 (70.1%) were male and 35 (30%) were female. The median age of the patients was 9.5 years (range: 46 years). The most common indications for stem cell transplant were beta-thalassemia major (BTM; n=58, 49%) and aplastic anemia (AA; n=42, 35.8%). However, the outcomes in match and mismatch groups showed significant results for positive direct Coombs test (DCT), indicating the occurrence of hemolysis. Despite the increased need for blood transfusions, ABO blood group incompatibility (ABOi) had no negative impact on the clinical results.

Conclusion

Based on our findings, ABO incompatibility does not affect the outcomes in patients undergoing alloHSCT. Patient monitoring can aid in early detection and treatment, thereby minimizing the frequency of fatal events.

## Introduction

Allogeneic hematopoietic stem cell transplantation (alloHSCT) is a curative treatment for a number of hematological illnesses, both malignant and non-malignant [1]. AlloHSCT, in contrast to organ transplantation, is more easily accomplished across the ABO blood group barrier [2-4]. It is unclear whether ABO mismatch has any effect on the clinical outcomes after allogeneic peripheral blood and bone marrow (BM) transplantation; however, the outcome of alloHSCT has improved significantly in the past few years. This has been achieved through advances pertaining to the selection of the donor, source of hematopoietic stem cells, supportive measures, creation of less toxic conditioning regimens, and access to novel post-transplant treatments. Globally, this advancement has widened indications for alloHSCT, with a corresponding increase in the number of transplant-eligible patients [5-7].

ABO incompatibility includes major, minor, or bidirectional types, and because of donor-recipient ABO blood group incompatibility (ABOi), alloHSCT is not restricted. This is seen in about 40-50% of alloHSCT procedures [3,4]. ABOi has been linked to various issues, including immediate and delayed hemolytic transfusion responses, pure red cell aplasia (PRCA), delayed engraftment, and a higher risk of developing acute graft-versus-host disease (GVHD) [8-11]; however, certain other studies have not confirmed this [5,12,13]. When it comes to the prevalence of GVHD, studies have often reported conflicting outcomes [14,15]. The influence of ABOi on transplantations in terms of neutrophil or platelet engraftment, non-relapse mortality (NRM), disease-free survival, and overall survival appears to be uncertain [4-8]. In the current study, we illustrate our experience in terms of the outcomes and complications observed due to ABO compatibility and incompatibility in hematopoietic stem cell transplantation (HSCT) performed for various hematological disorders at our center.

## Materials and methods

We conducted a retrospective, single-center, cohort study at the Institute of Blood Diseases and Bone Marrow Transplantation, Karachi. Patients were recruited for a period of three years from 2017 to 2019 and observed for 100 days post-transplant. Patients with hematological diseases such as beta-thalassemia major (BTM), Fanconi anemia, Gaucher disease, aplastic anemia (AA), acute myeloid leukemia (AML), and those undergoing a related full match and haploidentical alloHSCT at the National Institute of Blood Diseases and Bone Marrow Transplantation were included. Ethical approval was obtained from the Institutional Review Board of the National Institute of Blood Diseases and Bone Marrow Transplantation with approval no. NIBD/RD-196/07-2019. All methods were carried out in accordance with relevant guidelines and regulations of the Declaration of Helsinki. All the subjects gave their informed consent.

The most important factor in the blood group systems is to ensure ABO compatibility between the donor and patient; the ABO iso-group (match) refers to donors and patients having the same ABO blood groups. The major ABO-incompatible group has iso-agglutinins directed against donor red blood cell antigens from type A, type AB, or type B donor to type O recipient, whereas the minor ABO-incompatible group has iso-agglutinins against patient red blood cell antigens from type O donor to type A, type B, or type AB recipient. When iso-agglutinins against both donor and patient red blood cell antigens exist, it is referred to as bidirectional ABO incompatibility, type A donor to a type B recipient. Isoagglutinin titers were checked in the ABO-mismatch group to prevent hemolytic reactions. In case of a major mismatch and a recipient anti-donor isoagglutinin titer ≥1:32, the red cell in the peripheral blood stem cells (PBSC) was kept to <20 mL and plasma was diluted with FFP transfusions for four days to optimize the titer in the recipient to ≤1:32; but if the recipient had low titers, no manipulation of the graft was done.

Similarly, in minor ABO incompatibility and a high donor anti-recipient isoagglutinin titer (≥1:256), plasma depletion of both PBSC and BM grafts was performed. If the donor anti-recipient titer was low (≤1:128), no manipulation of the graft was done. In the case of bidirectional ABO incompatibility and high titers of anti-recipient isoagglutinin, both plasma depletion and recipient’s plasma were diluted with FFP for four days to optimize the titer. For hemolysis, the major group was assessed on days 0 and +1, for minor on days +5 and +15, and for bidirectional on days 0, +1, +5, and +15 during 100 days of follow-up. Direct Coombs test (DCT) was performed at baseline along with complete blood counts (CBC), serum lactate dehydrogenase (LDH), and liver function tests (LFTs). Hemolysis was identified through either increased LDH and/or indirect bilirubin levels that were more than twice the upper limit of the normal range. Reticulocyte count was observed on days +14, +21, and +28 to determine early transplant rejection. The Glucksberg-Seattle criteria [16] were used to classify acute GVHD, which was evaluated within the first 100 days of transplantation.

Moreover, neutrophil count equal to or greater than 0.5 × 10^9^ cells/L in the first three consecutive days or within the first 30 days of post-transplant was considered neutrophil engraftment, whereas platelets being equal to or more than 20 × 10^9^/L without transfusion assistance in the first seven days was deemed platelet engraftment. Transplant-related mortality (TRM) associated with alloHSCT problems that were not related to relapse was also tracked for 100 days after the transplant. Transplant-related complications were observed as PRCA or immediate hemolysis in the major mismatch group at days +0 and +1, whereas passenger lymphocytes were assessed with delayed hemolysis in the minor mismatch group as well as bidirectional mismatch group; moreover, delayed, immediate hemolysis, and PRCA were also assessed in bidirectional mismatch group for 100 days.

Statistical analysis

The normality of analytical variables was statistically significant on the Shapiro-Wilk test. Based on normality, non-parametric statistical analysis was performed. Descriptive analysis, i.e., qualitative, categorical variables at ordinal scale, included frequency in percent, weight, HLA typing, source of stem cells, diseases, blood groups, DCT, and chimerism with respect to both the patient and donor. Median and range were used for quantitative, continuous variables on the nominal scale, including age and laboratory parameters like LDH, indirect bilirubin, hemoglobin, and total leukocyte count. Wilcoxon test was employed to compare the pre- and post-transplant lab parameters. The Kruskal-Wallis test was used to evaluate the outcome variables of ABO-match and mismatch groups. The Cox regression technique was used to determine survival function for 100 days of follow-up with positive DCT. Statistical analysis was performed by using SPSS Statistics version 23.0 (IBM Corp., Armonk, NY). A p-value ≤0.05 was considered statistically significant.

## Results

A total of 117 allogenic transplant patients were recruited in the study, out of which 82 (70.1%) were males and 35 (29.9%) were females; the median age of the cohort was 9.5 years (range: 46 years), while the median age of the donors was 14 years (range: 51.5 years). Demographic characteristics of patients and donors are presented in Table 1. Patients were classified into two groups based on ABO compatibility; the match group comprised 75 patients, while the mismatch group had 42 patients. The mismatch group was further subdivided into major (n=17), minor (n=17), and bidirectional (n=8) groups. The range of the median dose of CD34-positive cells infused was 7.2 vs. 17.3 in ABO-compatible and incompatible groups respectively. Iso-hemagglutinin titers were elevated in six (35.5%) patients of the major mismatch group for which the patients' plasma was diluted with FFP transfusions to optimize the titer, while three patients (37.5%) required diluted plasma and plasma depletion in the bidirectional group, whereas no manipulation was done in the minor mismatch group. PRCA developed in two (50%) patients in the major and bidirectional groups respectively, whereas passenger lymphocyte syndrome (PLS) developed in two (66.7%) patients of the minor mismatch group and one (3.3%) patient in the bidirectional group developed these complications during the follow-up period spanning 100 days post-transplant.

**Table 1:** Demographic characteristics of patients and donors MRD: match-related donor

Allogeneic hematopoietic stem cell transplant (N=117)		
Demographic characteristics	Patient	Donor
Gender, n (%)		
Male	82 (70.1)	62 (53)
Female	35 (29.9)	55 (47)
Age group, n (%)		
≤18 years	77 (65.8)	74 (63.2)
>18 years	23 (19.7)	43 (36.8)
Allo transplant type, n (%)		
Haploidentical	36 (20.8)	-
MRD	81 (69.2)	-
Source of stem cells, n (%)		
Peripheral blood	-	58 (49.6)
Bone marrow	-	55 (47.)
Peripheral + bone marrow	-	4 (3.4)
Blood groups, n (%)		
A+	21 (17.9)	29 (24.8)
B+	29 (24.8)	32 (27.4)
O+	47 (40.2)	40 (34.2)
A-	1 (0.9)	1 (0.9)
B-	1 (0.9)	2 (1.7)
O-	2 (1.7)	3 (2.6)
AB+	15 (12.8)	9 (7.7)
AB-	1 (0.9)	1 (0.9)
Primary diagnosis, n (%)		
Beta-thalassemia major	55 (49.6)	-
Aplastic anemia	42 (35.9)	-
Acute myeloid leukemia	4 (3.4)	-
Severe combined immunodeficiency	5 (4.3)	-
Gaucher disease	2 (1.7)	-
Chronic myeloid leukemia	1 (0.9)	-
Paroxysmal nocturnal hemoglobinuria	1 (0.9)	-
Fanconi’s anemia	2 (1.7)	-
Acute lymphoblastic leukemia	2 (1.7)	-
Conditioning regime, n (%)		
Myeloablative	80 (68.4)	-
Non-myeloablative	37 (31.6)	-
Laboratory parameters		
Lactate dehydrogenase, µ/L	365 (1654)	-
Indirect bilirubin, mg/dl	0.36 (1.77)	-
Hemoglobin, g/dl	8.7 (9.8)	-
Total leukocyte count, × 10^9^/L	1.10 (89.9)	-
Platelets, × 10^9^/L	99.0 (816.0)	-

A comparison of laboratory parameters between pre- and post-transplant of ABO-match and mismatch groups showed a highly significant association with hemoglobin, platelet counts, total leukocyte counts, and total bilirubin respectively (p=0.000).

**Figure 1:**
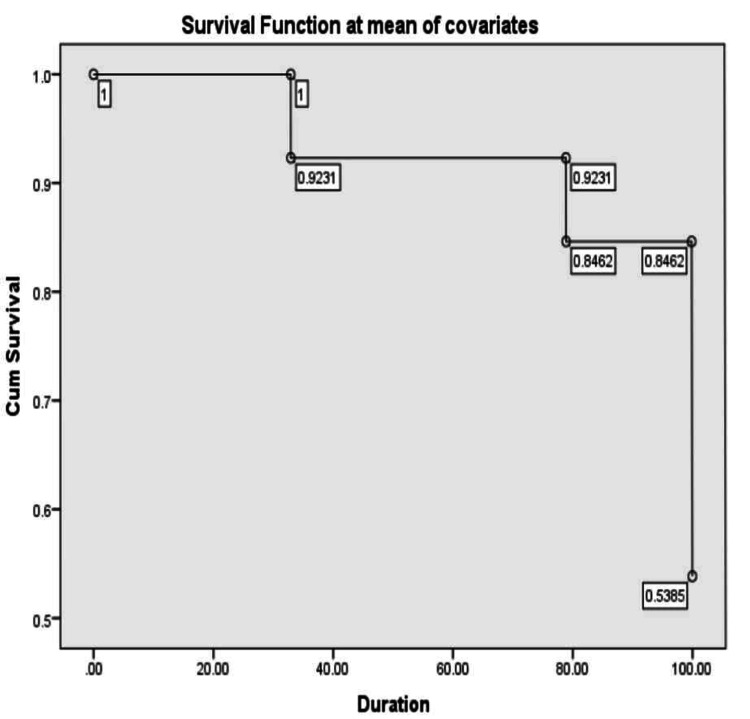
Survival function of major mismatch group

**Figure 2:**
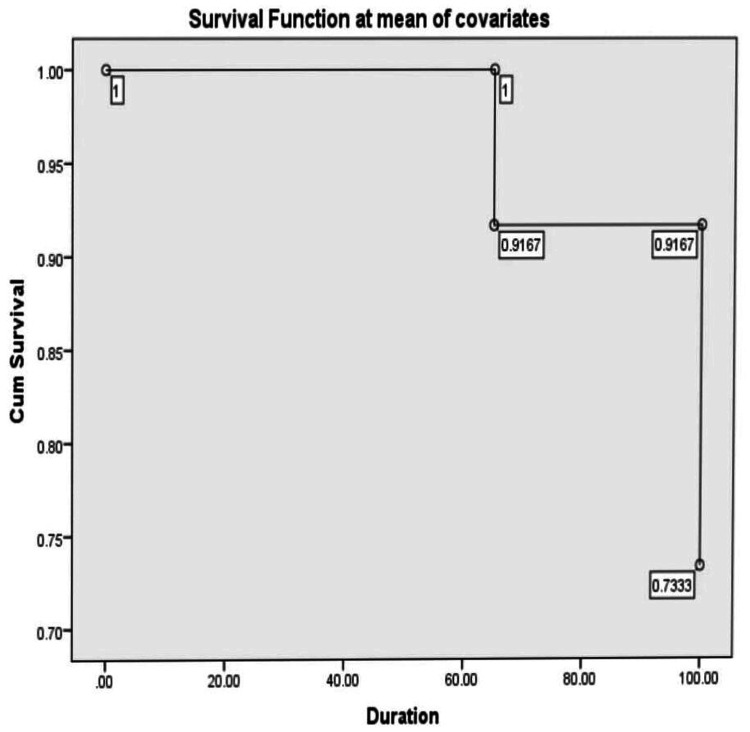
Survival function of minor mismatch group

**Figure 3:**
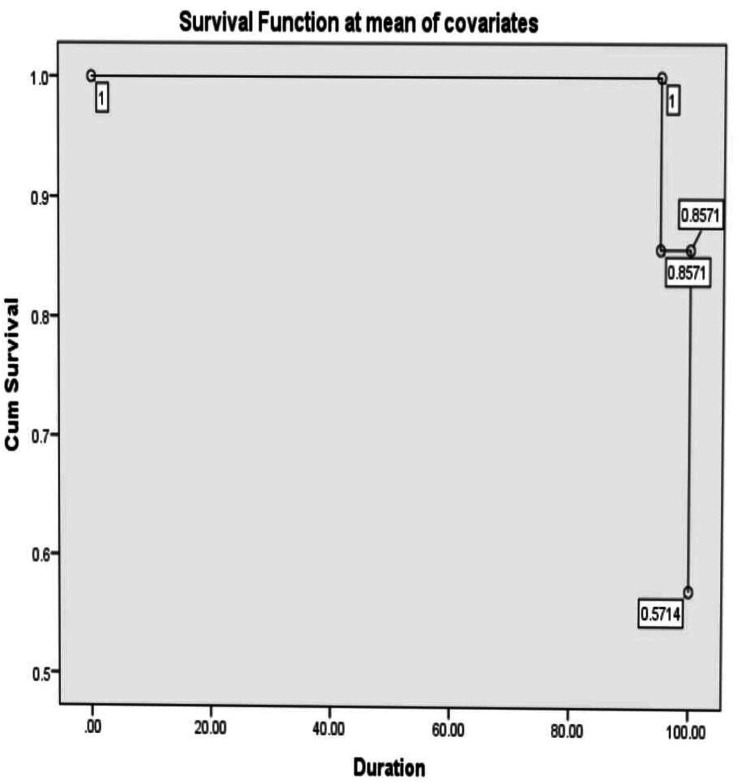
Survival function of bidirectional mismatch group

During the post-transplant period, hemolytic anemia was measured via retic count (%) in both groups. In the compatible group, the median value on day +14 was 0.96 (range: 61.8), on day +21 was 0.99 (range: 95.9), and on day +28 was 1.8 (range: 96); in the ABO incompatible group, it was 1.8 (range: 12.9) on day +14, 0.95 (range: 55.7) on day +21, and 1.65 (range: 31.18) on day +28 respectively. The association of outcome variables in the ABO-match and mismatch groups is displayed in Table 2, where a significant result was observed with positive DCT. High survival function was observed in the bidirectional and major mismatch groups: 87% and 76.5% respectively when compared to the minor group (70.6%), as shown in Figures 1-3. Donor chimerism was observed to be <5 in four (3.4%), <50 in two (1.7%), and >90 in 111 (94.9%) patients for the assessment of the risk of relapse after allogeneic stem cell transplantation. TRM showed insignificant results (p=0.785) with 12 (16%) deaths in the match group, while five (12.2%) were reported in the mismatch group.

**Table 2:** Outcome variables of ABO-match and mismatch groups *Statistically significant DCT: direct Coombs test; GVHD: graft versus host disease

Outcome variables	ABO match (n=75)	ABO mismatch (n=42)						
	Match	Major (n=17)	Minor (n=17)		Bidirectional (n=8)		P-value	
Engraftment, median (range)								
Platelets	11 (30)	12 (26)	14 (22)	12 (16)				0.967
Neutrophils	11 (21)	13 (21)	12 (19)	12 (8)				0.301
Transfusion, median (range)								
Platelets	18 (10)	11 (18)	12 (8)	1 (174)				0.465
Packed red cells	3 (13)	3 (2)	1 (3)	4 (8)				0.425
DCT, n (%)								
Positive	1 (7.1)	6 (46.2)	3 (23.1)	3 (23.1)				0.001*
Post-transplant complications, median (range)								
Pure red cell aplasia	-	2 (50)	-	2 (50)				0.128
Passenger lymphocyte syndrome	-	-	2 (66.7)	1 (33.3)				0.333
GVHD, n (%)								
Skin	4 (57.9)	2 (28.6)	1 (14.3)	-				0.548
Gut	9 (56.3)	2 (12.6)	4 (25)	1 (6.3)				0.616
Liver	4 (57.1)	1 (14.3)	1 (14.3)	1 (14.3)				0.808
Oral	9 (75)	1 (8.3)	1 (8.3)	1 (8.3)				0.808
Ocular	2 (100)	-	-	-				0.716
Graft failure, n (%)								
Primary	8 (72.7)	3 (27.3)	-	-				0.251
Secondary	5 (71.4)	6 (8)	1 (5.9)	1 (5.9)				0.861

## Discussion

The goal of this single-center study was to assess the effect of donor/recipient ABO blood groups on transplant outcomes in a large cohort of patients. This is, to the best of our knowledge, one of the largest local studies to be ever conducted. In Pakistan, the common hematological diseases that affect our population are benign diseases such as BTM, acquired AA, bone marrow failure syndromes, and immunodeficiency states followed by acute leukemias [17]. The only curative treatment for these disorders is HSCT. At present, only match-related donor transplants are performed in Pakistan, and other stem cell donation programs do not exist. In nearly half of all cases, a large family size proves to be beneficial in terms of providing a match-related donor (e.g., siblings, parents, and extended family members). The transplant activity is likely to increase due to recent developments in haploidentical HSCT. So far, 36 haploidentical transplants have been carried out at our center with promising results.

The majority of patients in this study were less than 18 years of age (65.8%), with the primary indications for stem cell transplants being benign diseases. In Pakistan, an estimated 5000-9000 children are born each year with BTM and it remains a major health and socioeconomic issue for the families and the country at large [18]. The treatment options for this condition are limited: transfusion or transplantation. In the last two decades, with the establishment of transfusion facilities in the country, acquisition of relevant knowledge, and training of trainers in stem cell transplant, nine transplant centers were accredited by the national legislature body and approximately 2800 HSCTs have been carried out, predominantly for benign disorders [19,20]. AA is uncommon in European and North American populations, but it is common in Asian populations [21]. The precise cause of AA is unknown. Genetic and environmental causes have been proposed in various hypotheses [21].

There is no data on the prevalence of AA in Pakistan, although numerous studies conducted locally have reported a high prevalence of this condition (recently estimated to be approximately 3.5 patients/million people based on data gathered from all of the country's specialized medical care institutes) [22]. A large case series study of 1324 AA patients identified 489 (37%) patients aged 15-24 years, followed by 355 (26.8%) aged <15 years; these findings are similar to our study, where the majority of the patients were less than 18 years of age (65.8%), while only 19.7% were older than 18 years. This high prevalence in younger age groups can be attributed in part to the demography of the country, with 19.3% and 34.7% of the population falling into the age groups of 15-24 years and <15 years respectively [23]. Male patients predominated in Asian countries according to studies [24]. Our cohort had a male-to-female ratio of 2.3:1, which aligns with previous studies from the region [24-26]. The apparent high number of males in our study (70.1%) could be due to social biases since men are more valued in our society and hence receive preferential treatment in terms of medical care.

In our study, a total of 117 patients had HSCT, and they were classified into two groups. Platelets, red cells, and neutrophil engraftment in ABO-compatible and incompatible groups were not significant. However, the median number of platelets and red cell transfusions showed higher values in the incompatible group as compared to the compatible group. Moreover, no statistically significant differences were found in terms of complications (Table 2) or survival between the ABO groups, as indicated in Figures 1-3. Four patients had PRCA in the major and bidirectional groups respectively. This was confirmed on bone marrow biopsy and treated with high-dose steroids. However, two patients needed rituximab injections for the resolution of PRCA apart from increased red cell transfusions. Similarly, three patients with PLS were treated with steroids and none of the patients required therapeutic plasma exchange.

The effect of ABO incompatibility on the clinical outcomes following HSCT has been studied, and it has no influence on patient survival or TRM according to a study conducted by the Center for International Blood and Marrow Transplantation; nevertheless, the duration of PRBC transfusion was prolonged in the major ABO-incompatible group [6]. However, other studies have found that ABO incompatibility might have an effect on clinical outcomes and can occasionally lead to severe hemolytic consequences. Nonetheless, the findings have been contradictory, and the patient-related information/data and materials used in these studies have been diverse [27-29]. The results of ABO match vs. mismatch on hematopoietic reconstitution revealed no significant impact. Similarly, our study demonstrated no effect of significant ABO incompatibility on neutrophil or platelet recovery independent of donor type, conditioning regimen, or stem cell source except for increased platelet and red cell transfusions. These findings are consistent with prior research, which found that substantial ABO mismatch had little effect on engraftment [30].

This study has a few limitations. It was conducted at a single center and employed a retrospective design. Even though it involved a substantial number of patients, the sample size may still be insufficient for detecting more subtle associations or rare outcomes, potentially affecting the statistical power of the analysis. In this investigation, we found no unfavorable effect of hemolysis over the first 100 days following transplantation. Furthermore, high survival function was reported in the bidirectional and major mismatch groups: 87% and 76.5% respectively compared to the minor group (70.6%) by applying Cox regression analysis. None of these individuals experienced clinically substantial hemolysis, as previously documented by others following ABO-incompatible transplantation [9]. Acute GVHD, which develops in identical proportions in patients transplanted with either source of HSC, hastens the resolution of these iso-hemagglutinins [14,15].

In our study, we did not find any significant differences with regard to the impact of the stem cell sources - whether PBSCs or BM - on engraftment and GVHD. Following transplantation, most of our patients received a two-drug prophylactic regimen consisting of methotrexate and cyclosporin or mycophenolate mofetil. Methotrexate and mycophenolate mofetil are anti-proliferative agents that may inhibit the proliferation of donor lymphocytes carried in stem cell inoculums; thus, our findings do not suggest that using PBSC as a source of HSC increases the risk of delayed hemolysis and complications after minor ABO-incompatible transplantations.

## Conclusions

Based on our findings, ABO incompatibility appears to have no effect on the clinical outcomes or survival in patients undergoing alloHSCT. In this study, patients in the ABO-mismatch group required more packed red cells and platelet transfusions following HSCT than those in the ABO-match group. Despite the greater need for blood transfusions, no significant differences in the clinical outcomes were observed between the ABO groups. Careful patient monitoring can help in detecting and managing issues early, thereby minimizing the number of life-threatening events. However, more research is needed to gain deeper insights into this immunological mechanism because ABO incompatibility was not found to affect the clinical outcomes. Multicenter studies involving a larger number of patients with ABOi may help to more comprehensively understand the effects of ABOi on the outcomes, especially in our population.
